# Development of an eHealth tool for cancer patients: monitoring psycho-emotional aspects with the Family Resilience (FaRe) Questionnaire

**DOI:** 10.3332/ecancer.2018.852

**Published:** 2018-07-11

**Authors:** Flavia Faccio, Chiara Renzi, Chiara Crico, Eleni Kazantzaki, Haridimos Kondylakis, Lefteris Koumakis, Kostas Marias, Gabriella Pravettoni

**Affiliations:** 1 Department of Oncology and Hemato-Oncology, University of Milan, Via Festa del Perdono 7, Milano 20122, Italy; 2 Applied Research Division for Cognitive and Psychological Science, European Institute of Oncology, Via Ripamonti 435, Milano 20141, Italy; 3 Computational BioMedicine Laboratory, FORTH-ICS, Heraklion 70013, Crete, Greece; * These authors equally contributed to the manuscript.

**Keywords:** resilience, psychological, family health, telemedicine, neoplasms, health services

## Abstract

In the last decade, clinicians have started to shift from an individualistic perspective of the patient towards family-centred models of care, due to the increasing evidence from research and clinical practice of the crucial role of significant others in determining the patient's adjustment to cancer disease and management. eHealth tools can be considered a means to compensate the services gap and support outpatient care flows. Within the works of the European H2020 iManageCancer project, a review of the literature in the field of family resilience was conducted, in order to determine how to monitor the patient and his/her family's resilience through an eHealth platform. An analysis of existing family resilience questionnaires suggested that no measure was appropriate for cancer patients and their families. For this reason, a new family resilience questionnaire (named FaRe) was developed to screen the patient's and caregiver's psycho-emotional resources. Composed of 24 items, it is divided into four subscales: Communication and Cohesion, Perceived Family Coping, Religiousness and Spirituality, and Perceived Social Support. Embedded in the iManageCancer eHealth platform, it allows users and clinicians to monitor the patient's and the caregivers' resilience throughout the cancer trajectory.

## Introduction

Outpatient care flows are a common modality in oncology settings and they consist of short encounters at the clinic for medical consultation and/or therapy administration [1–4], which can take place several days or weeks from one another. During these appointments, the involvement of patients and their families in the medical decision process of treatment plans is increasing [5]. The patient together with the clinician works towards increased awareness of personal resources, which can prevent the onset of psychological exhaustion. Clinicians are moving towards family-centred models of care as significant others’ coping strategies can determine adjustment and overall wellbeing of the patient and those that surround him/her. In the past, the process of empowering patients and increasing their self-efficacy in managing the disease was often implemented during face-to-face encounters at the clinic. However, this support was provided only at an individual level, as the family network of the patient was considered only in terms of its deficits and weakness; nowadays, the clinician focuses on the family’s strengths and resources, which can activate shared resilience processes [6]. Throughout the illness trajectories, patients and their families often express the need to receive reliable, sometimes extensive health-related information and counselling for decision-making. They also often ask for emotional and psycho-social support; however, screening for psychological distress within clinical consultations is often inadequate or insufficient due to various reasons such as lack of resources of the healthcare systems and stigma or privacy concerns of patients [7, 8]. As a consequence, it may not always be possible to provide high quality and efficient self-management support through the standard care flows. For this reason, it is vital to develop and implement psychological evidence-based interventions for cancer patients and their caregivers, keeping in mind the trajectory and the phases of the illness [9].

Information about the impact of the disease on the family, on the relationship with the partner, and their coping strategies represent some of the most common unmet needs for family members of a patient with cancer [10]. Caregivers frequently request psychological support, particularly for fears regarding the patient’s disease progression or recurrence [10, 11]. It is estimated that around 40% of caregivers of cancer patients suffer from depression and anxiety [10], 53% experience moderate or severe fatigue [11] and up to 95% are affected by moderate to severe sleep disturbances [12]. Financial burden represents another important aspect, with estimated costs ranging from $31,000 to more than $91,000 [13]. Studies have also reported changes in caregivers’ physical health, with high caregiving burden representing an independent risk factor for coronary heart disease and death from other causes [14]. Family quality of life after a cancer diagnosis is predicted by illness survival stressors (such as concurrent family stressors and fear of recurrence), social support, and family meaning of cancer illness [15–17]. Patient and caregiver psychological distress were interdependent in two meta-analyses, the emotional well-being of one family member affecting the others’ [18, 19]. Although interventions for patients and caregivers of cancer patients have shown a reduction in the frequency of negative effects, improved coping skills, and quality of life [20], these are rarely implemented due to lack of awareness, lack of professional training, insufficient professional time and costs of implementation [21].

To compensate for the gap of professional help, web-based tools were identified as a promising means to empower cancer patients and survivors [22–26] and satisfy their supportive needs [27, 28]. Patients often consult Internet resources and search for support on web platforms [29, 30]. A recent study assessing supportive care needs and attitudes towards eHealth in an online sample of cancer patients pointed out that psychological support is one of most frequently expressed needs [31], confirming the need for increased online encounters.

In this perspective, eHealth may represent a way to respond more broadly to these needs, in light of the fact that more and more individuals with cancer are facing important treatment decisions, emotional distress and physical challenges due to the disease or treatment plan.

Taking these premises into account, within the works of the European H2020 project iManageCancer (grant agreement no. 643529) to develop an eHealth platform composed by apps and services to improve self-management and to promote cancer patients’ empowerment, psycho-social monitoring tools were included. The aim of these tools is to provide fast and effective screening of emotional, cognitive and social functioning and to provide self-management support and appropriate referral to specialists [21–23]. In particular, a novel aspect that was considered in the development of these services is the systemic framework, as the crucial influence of significant relationships with others is now considered an essential contributing factor of an individual’s potential to adjust and manage cancer-related challenges [9, 35]. In the last decade, a psychological construct which has received great interest and which encompasses the dynamic psycho-emotional processes and resources of the support network is family resilience.

The rest of this paper is structured as follows: In the section ‘Literature review’, we review the literature and present similar approaches, identifying their drawbacks. Then, in the section ‘Developing a questionnaire to assess family resilience’, we present the questionnaire developed in the context of iManageCancer. In the section ‘Implementation of the FaRe Questionnaire in the platform’, we present the implementation of the tool and finally, the section ‘Conclusion’ concludes this paper and presents directions for future work.

## Literature review

Family resilience refers to the system’s ability to withstand stressful experiences and rebound from these by creating a new, healthy family functioning [36]. It encompasses multiple strengths and resources, which are considered dynamic processes that reduce the risk of functioning in a maladaptive way, support adaptation to the stressor, and provide ground for potential personal and relational growth. Walsh [36, 37] identifies key processes that can help families overcome challenges while taking into account the uniqueness of each family unit. Individual resilience can be understood and fostered within the family context and all individuals can potentially increase their resilience. Focusing on the family’s strengths rather than its deficits and assessing the functioning within each family context, considering its values and resources, can give a novel and more useful insights to support this system throughout the cancer phases.

During the preliminary activities for the development of these services, a revision of the literature was conducted in order to assess which measures are currently available to evaluate family resilience. As this field of research is relatively new compared to individual resilience the development of measurement scales began just over a decade ago [25, 26, 38–41].

Three questionnaires have attempted to capture the dynamic processes that allow families to respond positively to a stressful event. Walsh [39] developed a self-report questionnaire, the Walsh family resilience questionnaire, composed of 33 items, divided into the nine key processes of her family resilience model [36]. According to the author, this instrument should be used to evaluate the effectiveness of family-oriented interventions and to assess resilience pre- and post-support. Nevertheless, data from studies applying this questionnaire in diverse contexts have not been published yet.

Sixbey [41] instead developed a 54-item measure, the Family Resilience Assessment Scale (FRAS), on a four-point Likert scale, with higher scores indicating higher levels of resilience. Sixbey [41] divided the measure into six subscales: (i) family communication and problem-solving, (ii) social and economic resources, (iii) maintaining a positive outlook, (iv) family connectedness, (v) family spirituality and (vi) making meaning of adversity. The questionnaire was administered to the general American population without considering an essential aspect of resilience, namely whether an adversity had occurred. The main limitation of the FRAS is the disagreement between developing authors and subsequent studies on the number of factors and therefore which key processes of family resilience are present in the scale. The FRAS has been recently applied in healthcare contexts to families in a sample of epileptic young adults [42].

Another measure developed according to Walsh’s [36] framework is the Family Resilience Assessment (FRA) [38]. This 29-item measure, divided into three subscales that resemble Walsh’s [36] three overarching constructs, was developed for women with a history of breast cancer. Some important limitations of the FRA need to be addressed: first, its focus is on the individual’s experience of cancer rather than the family’s perspective, lacking a systemic and ecological view of the family. Second, the FRA has not been used in other oncological or more general health contexts, suggesting the need for further studies in order to consider the instrument a valid and reliable measure of family resilience.

## Developing a questionnaire to assess family resilience

To date, there is only one measure of family resilience in cancer patients, namely the validation of the FRA in women with breast cancer, [38] which has an individualistic view of resilience and does not capture the systemic processes involved.

For this reason, a new measure was developed to address family resources and strengths in the management of cancer taking into account also the family’s perspective. Developed on the basis of Walsh’s [36] model, the main aim of the FaRe Questionnaire is to assess both the patients’ and the caregivers’ resilience. To our knowledge, this is the first tool to actually implement a systemic perspective in an oncology setting. The questionnaire can be filled in both by patients and caregivers, and their scores can be compared, looking for similarities or differences across different domains. It highlights the critical areas that can deplete the family’s resources as well as identify their strengths, which can be considered a starting point to develop a tailored intervention. The possibility to embed this tool in a web platform allows not only the assessment of family resilience but also the ability to monitor it at any desired moment throughout the cancer phases. It provides patients and family members with a practical tool from which they can gain insights on their wellbeing and ‘legitimises’ the requests of support to specialised professionals. On the other hand, it can provide clinicians and institutions with a monitoring tool that supports the implementation of efficient care flows.

The questionnaire was validated in a study conducted at the European Institute of Oncology with breast and prostate cancer patients and their caregivers. The works related to the validation of the questionnaire will be described in detail elsewhere (manuscript in preparation). The final questionnaire is composed of 24 items divided into four factors: Communication and Cohesion, Perceived Social Support, Perceived Family Coping, Religiousness and Spirituality.

For each factor, normative scoring of the population was provided together with feedback to patients and clinicians depending on the individual score obtained. Different profiles were prepared for scores above or below two standard deviations (SDs) from the mean population score; one profile with scores between 1 and 2 SDs above or below the mean population score; another profile with scores within the range of −1 and 1 SDs from the mean. These profiles can inform users about the meaning of the factor considered, providing a reference to scientific evidence explained in lay language and suggestions for possible self-management actions, including referral to clinicians in some cases.

## Implementation of the FaRe Questionnaire in the platform

**Figure 1.**
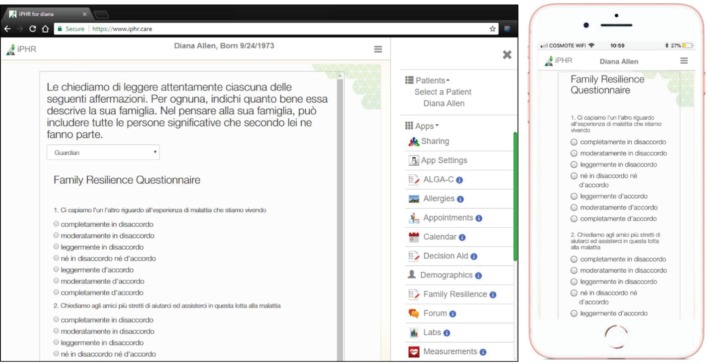
Screenshots of the FaRe tool using a computer (left) and a mobile device (right).

The FaRe Questionnaire was implemented as an app within a personal health record system (named iPHR) [31], accessible both via desktop computers as well as in mobile-friendly visualisation (Figure 1).

The questionnaire can be filled in by both patients and family members. The iPHR periodically reminds the user to perform the family resilience evaluation. All data are computed and stored by the tool depending on the reference population for the individual completing the questionnaire.

In the case of significantly elevated scores, an alert is also sent to clinicians, if the patient or the family member has authorised sharing of information with a healthcare professional. This reduces the distance between the medical reference figure and the patient, providing a bridge for communication, albeit through apps. In future implementations of the tool in a clinical context, clinicians could consider contacting the patient and proposing different types of interventions or the patient/family member could contact a healthcare professional. The platform could thus represent a tool to actively share information between members of the multidisciplinary team and of the family. In the next phase of the project, the FaRe tool will provide comparison scores between different members of the family and send an alert when significant discrepancies in global scores or single factors are registered. This valuable information can help to respond to the family’s needs, addressing possible internal crisis and miscommunications.

We expect that the tool will provide increased levels of awareness in the patient and the family members regarding their resources and the importance of a supportive family and social network in managing cancer-related issues. It will also allow healthcare professionals to stay in touch with the psychological dimension of the disease and to focus more on the importance of monitoring it throughout time, as different illness phases may be critical for different patients. In turn, we assume that referral to psychological services will be easier and more accessible; consequently, patients and their caregivers will perceive greater value in the type of care provided. The final expected outcomes of the use of the tool are a better adjustment to the disease and reduced probability of mental health problems, which in turn can hinder adherence and self-management.

## Conclusion

This paper focuses on the implementation of an eHealth tool developed to monitor family resilience through a questionnaire named FaRe. This tool aims to promote family engagement, provide recommendations that could increase empowerment and support self-management of all individuals involved in the cancer trajectory, alerting clinicians when patients may benefit from psychological support.

The enrolment of patients to evaluate the whole iManageCancer platform and, amongst this, the FaRe, has already started and 120 cancer patients and their families have used the tool for a period of at least 6 months. Analysis of its usability and effectiveness will be discussed in future works.

## Funding

**Figure:**
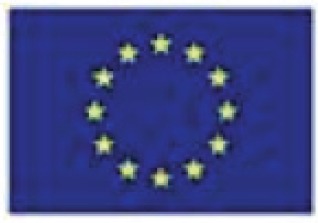

This project has received funding from the European Union’s Horizon 2020 research and innovation programme under grant agreement No. 643529.

## Disclaimer

This paper reflects the authors’ view. The Commission is not responsible for any use that may be made of the information it contains.
